# Multiple environmental changes drive forest floor vegetation in a temperate mountain forest

**DOI:** 10.1002/ece3.2801

**Published:** 2017-03-01

**Authors:** Norbert Helm, Franz Essl, Michael Mirtl, Thomas Dirnböck

**Affiliations:** ^1^ Division of Conservation, Vegetation and Landscape EcologyUniversity of ViennaViennaAustria; ^2^ Department for Ecosystem Research and Data Information ManagementEnvironment Agency AustriaViennaAustria

**Keywords:** acidification, climate change, disturbance, forest floor vegetation, LTER Zöbelboden, resurvey

## Abstract

Human‐induced changes of the environment and their possible impacts on temperate forest understory plant communities have been examined in many studies. However, the relative contribution of individual environmental factors to these changes in the herb layer is still unclear. In this study, we used vegetation survey data covering a time period of 21 years and collected from 143 permanent plots in the Northern Limestone Alps, Austria. Data on soil chemistry (49 plots), light condition (51 plots), soil temperature and moisture (four and six plots), disturbance (all plots), climate (one station in a clearing area), and airborne sulfur (S) and nitrogen (N) deposition (two forest stands) were available for analyses. We used these data together with plot mean Ellenberg indicator values in a path analysis to attribute their relative contributions to observed vegetation changes. Our analysis reveals a strong directional shift of the forest understory plant community. We found strong evidence for a recovery of the ground‐layer vegetation from acidification as response to decreased S deposition. We did not observe a community response to atmospheric N deposition, but we found a response to altered climatic conditions (thermophilization and drying). The path analysis revealed that changes in the light regime, which were related to small‐scale disturbances, had most influence on herb layer community shifts. Thermophilization and drying were identified as drivers of understory community changes independent of disturbance events.

## 1 Introduction

Human activities have led to fundamental alterations of the environment causing unprecedented and accelerating losses of biodiversity worldwide (MEA 2005; Tittensor et al., 2014). Among the many pressures to biodiversity, climate change, land use change, and excess nitrogen (N) deposition have emerged as the most important for biodiversity losses (Foley et al., 2005; MEA 2005; Pereira, Navarro, & Martins, 2012). Yet, their relative impacts on biodiversity are difficult to disentangle and vary across spatial and temporal scales (Bernhardt‐Römermann et al., 2015; Essl et al., 2015).

Species are differently affected by environmental forcing, and this in turn will affect the phenology, abundance, and distribution of species, as well as the interactions with other species (Hobbs et al., 2006; Parmesan, 2006; Peñuelas et al., 2013). With progressing climate change, the impacts of more frequent and intense extreme events (e.g. droughts, storms) and biotic disturbances (e.g. bark beetle outbreaks) are predicted to increase in forests (Seidl, Schelhaas, Rammer, & Verkerk, 2014; Weed, Ayres, & Hicke, 2013), with decisive consequences for biodiversity (Thom & Seidl, 2015). Land use changes, particularly the loss and fragmentation of forests, had strong negative impacts on global (Newbold et al., 2015; Pimm & Raven, 2000; Vié, Hilton‐Taylor, & Stuart, 2009) and European (Dupouey, Dambrine, Laffite, & Moares, 2002; Glatzel, 1990; Perring et al., 2016) forest biodiversity.

From the nineteenth century onward, N deposition mainly originating from fossil fuel combustion, agricultural fertilization, and livestock breeding has increased strongly and is presumed to be responsible for a substantial decline of plant species richness (Bobbink et al., 2010; Gilliam, 2006) and altered community structures in temperate forests (Meunier, Gundale, Sánchez, & Liess, 2016). Yet, the level of N deposition impacts strongly depends on site conditions as well as on the history and magnitude of deposition amounts (Bernhardt‐Römermann et al., 2015; Dirnböck et al., 2014). In industrialized countries, air pollution caused large‐scale forest soil acidification in the second half of the last century, but some recovery in soils (Akselsson, Hultberg, Karlsson, Pihl Karlsson, & Hellsten, 2013; Cools & De Vos, 2011) and forest vegetation (Reinecke, Klemm, & Heinken, 2014; Vanhellemont, 2014) has been observed after drastic declines in sulfur (S) emissions from the 1980s onward (Vet et al., 2014). According to the study of Vet et al. (2014), N deposition has recently also declined in many areas in Europe.

Forest understory vegetation has a prominent role as a major component of forest biodiversity and for ecosystem function (Gilliam, 2014). Yet, because of the complexity of forest ecosystems, it is particularly difficult to disentangle the impacts of multiple drivers on understory properties. Microclimate, soil chemistry, and irradiance are the main factors determining understory species composition (Leuschner, 1999; Leuschner & Lendzion, 2009). These factors differ dramatically between forests and they change with forest stand age (Leuschner & Rode, 1999) because tree composition and structure controls the microclimate at the forest floor (Geiger, Aron, & Todhunter, 2009; Norris, Hobson, & Ibisch, 2012), and because nutrient availability is determined by leaf litter quantity and quality (Lovett, Weathers, Arthur, & Schultz, 2004; Verheyen et al., 2012). As forest floor vegetation is so tightly related to characteristics of the overstory (Whitney & Foster, 1988), tree canopies and their characteristics have restrained or buffered climate change (Bertrand et al., 2011; De Frenne et al., 2013) and N deposition impacts (Verheyen et al., 2012) on the forest understory. However, disturbances may serve as trigger of more rapid changes of the forest understory (Thom et al., 2017).

Long‐term forest vegetation resurvey data are becoming increasingly available (Verheyen et al., 2017). This allows the study of compositional vegetation changes and to examine their possible environmental drivers. Some studies found evidence that forest floor vegetation has already responded to increasing temperatures (Küchler, Küchler, Bedolla, & Wohlgemuth, 2014; Lenoir, Gégout, Marquet, de Ruffray, & Brisse, 2008; Savage & Vellend, 2015). Several studies observed responses to increased soil N availability due to N deposition (Bernhardt‐Römermann et al., 2007; Brunet, Diekmann, & Falkengren‐Grerup, 1998; Diekmann, Brunet, Rühling, & Falkengren‐Grerup, 1999; Keith, Newton, Morecroft, Bealey, & Bullock, 2009; Naaf & Kolk, 2016; Reinecke et al., 2014; Thimonier, Dupouey, Bost, & Becker, 1994) and to acid rain (Diekmann et al., 1999; Hédl, 2004; Thimonier et al., 1994), while others report recovery from acidification in recent years (Reinecke et al., 2014; Vanhellemont, 2014). Further, local factors such as forest management, disturbance regime, and game density have been shown to be responsible for affecting the responses of the forest herb layer to deposition effects and climate warming (Bernhardt‐Römermann et al., 2015; Hédl, 2004; Hopkins & Kirby, 2007; Müllerová, Hédl, & Szabó, 2015).

Here, we present a resurvey study from the Northern Limestone Alps in Austria that analyzes the drivers of changes in forest understory composition over 21 years. We use data of 143 permanent plots which record data on vegetation, disturbance events, and browsing damage to detect changes in floristic species composition and the environment, partially represented by Ellenberg indicator values. Further, soil chemical and radiation data are available for 49 and 51 of these plots, respectively, soil temperature and moisture data for four and six of these plots, respectively. Macroclimatic data were retrieved from a climate station located in a clearing area at the plateau, and air deposition data have been recorded on two different sites in the study area. We analyze these measured environmental parameters regarding their temporal changes and compare them with the observed shifts in vegetation and Ellenberg indicator values.

**Figure 1:**
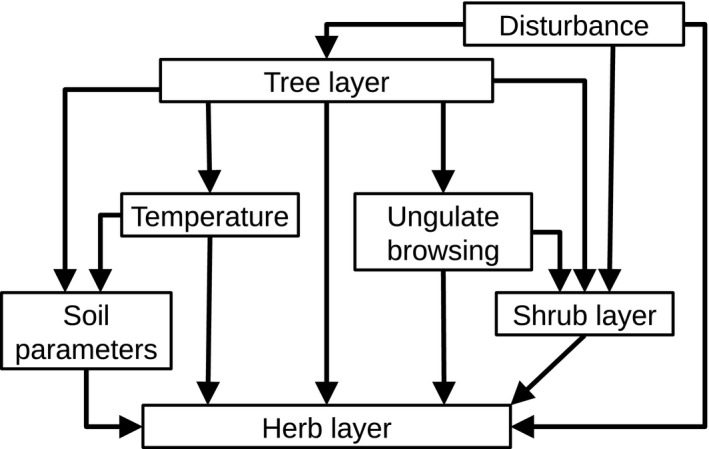
Conceptual model that was used for the path analysis. Boxes represent the different variables and arrows the hypothesized relationships among them: We assumed disturbance as the only factor controlling tree layer cover and also controlling shrub cover and directly affecting the herb layer via soil perturbations. The tree layer has a key role by influencing the microclimate and soil conditions, limiting the light availability for the understory and thus the development of the herb and the shrub layer. Ungulate browsing may differ between open and dense forests and impacts the shrub and the herb layer. Although some relationships may have reciprocal influence, we used only one‐directional pathways to avoid a too complex model

Specifically, we asked the following questions, which are based on the theoretical model in Figure 1: (1) What are the changes in environmental (e.g. climate, airborne nitrogen deposition) and biotic (e.g. browsing, changes in tree layer composition) drivers during the last two decades that potentially affected forest floor vegetation? (2) Which drivers did influence changes in the forest floor species composition and structure? Given changes in climate and deposition, we hypothesized that (i) disturbance had most impact on the species composition of the herb layer because it controls light regime in the forest understory, (ii) climate change favoured thermophilic and drought‐tolerant species, (iii) basophilic plant species increased and acidophilic decreased, and (iv) eutrophic plant species became more frequent. As to species diversity we hypothesized that (v) increasing disturbance increased species numbers in plots and that disturbance led to higher compositional differences among plots. We did not expect climate change effects on species diversity as immigration of thermophilic species should offset potential losses of cold‐tolerant species, nor did we expect that N deposition or soil recovery from acidification to have a significant effect because the study area is not strongly N limited and has a high buffer capacity for acid deposition.

## 2 Methods

### 2.1 Study area

The study site “LTER Zöbelboden” is located in the National Park Kalkalpen in Upper Austria (47°50′30″N, 14°26′30″E). It was established in 1992 by the Environment Agency Austria as part of the effects monitoring network within the UN Convention on Long‐Range Transboundary Air Pollution (CLRTAP), and it became part of the Austrian Long‐term Ecosystem Research Network (LTER). In the southern and eastern section of the study area, a plateau stretches at about 900 m a.s.l. while the remaining part of the study area consists of steep, north‐, west‐, and east‐facing slopes, forming the 90 ha catchment of Zöbelgraben with the lowest elevational point on 550 m a.s.l. The main bedrock is dolomite, soils on the slopes are mainly lithic and rendzic leptosols, whereas on the plateau chromic cambisols and hygromorphic stagnosols dominate (Hülber et al., 2008). Most of the study area is covered by a near‐natural temperate deciduous forest with European beech (*Fagus sylvatica* L.), sycamore (*Acer pseudoplatanus* L.), ash (*Fraxinus excelsior* L.), other deciduous tree species and the conifers Norway spruce (*Picea abies* Karst.), larch (*Larix decidua* Mill.), and fir (*Abies alba* Mill.), whereas on the plateau remnants of a spruce plantation of the early twentieth century can be found. Since 1992, forest management has been restricted to the removal of bark beetle‐infested trees. Since 2004, the area has been hit by several storms causing windthrow and subsequent bark beetle infestation of Norway spruce. The mean annual temperature (1980–2012) at Zöbelboden is 7.4°C (measured in 900 m a.s.l.). The warmest month is July (15.3°C), and the coldest month is January (−2.0°C). Average annual precipitation is 1628 mm and interannual variation ranges from 1400 to 1800 mm (1990–2013), with the highest precipitation sums in July (198 mm) and lowest in February (101 mm). Snow cover usually prevails until March.

### 2.2 Sampling design

#### 2.2.1 Vegetation data

Over the whole study area, a rectangular grid of 100 by 100 m with 10 × 10 m permanent plots at every grid corner and the middle points (totaling to 165, excluding rock outcrops, and young plantations of less than 20 years) was established in 1993. Vegetation resurveys were made in 2005, 2010, and 2014. Twenty‐two of the 165 plots could not be relocated in every survey year, as the corner marks were temporarily destroyed by slope movements or uprooting of trees. They had to be excluded from some of the resurveys causing a reduction to 143 plots when comparing all four resurvey years. A total of 154 plots were surveyed in 2014 and 1993 and could be used for the analyses comparing only these two survey years. Surveys were carried out during the main growing season (July, August) to avoid phenological bias. All vascular plant species occurring at the plots were recorded, and their cover was estimated in seven cover‐abundance classes according to Braun‐Blanquet (1964). Woody species were assigned to five different vegetation strata depending on their height: herb layer (0–60 cm), shrub layer (60–300 cm), tree layer 3, tree layer 2, and tree layer 1.

To eliminate errors of misidentification, we pooled the following closely related and taxonomically difficult species: *Senecio ovatus* Willd. and *S. nemorensis* L. (to *Senecio nemorensis* agg), *Dryopteris carthusiana* H.P.Fuchs and *D. dilatata* A.Gray (to *Dryopteris carthusiana agg*.). Bryophytes and lichens were excluded from the analyses. For analyses, we transformed all Braun‐Blanquet cover values to ordinal values (1–7).

#### 2.2.2 Ungulate browsing damage

To measure the damage of ungulate browsing, we selected the six tallest individuals of each tree species within reach of ungulates (maximum 130 cm) and noted whether the main trunk showed damage by ungulate browsing in the year of the record, in the preceding year or both years, and calculated the percentage of damaged individuals. Measurements for ungulate browsing damage were carried out in the resurvey years 2005, 2010, and 2014.

#### 2.2.3 Tree layer cover

During the vegetation surveys, the total cover of the three tree layers and the shrub layer were estimated (in %). For each survey year and each plot, we later calculated the total tree cover by adding the cover values of the tree layers 3, 2, and 1.

#### 2.2.4 Disturbance data

Areas affected by disturbances from windthrow or spruce bark beetle outbreaks between the first and the last survey year were identified based on aerial photographs (years 1994, 2000, 2003, 2008, 2009) and field survey records (at least every 2 years). Every plot located within a disturbance patch or where individual trees (from the tree layer 1 or 2) within the 5‐m surrounding area of the 10 × 10 m plots fell or died due to windthrow or bark beetle were deemed disturbed.

#### 2.2.5 Soil data

We collected mixed soil samples at a subset of 49 permanent plots in July and August 2014 for comparison with soil data from 1992 and 2004. Sampling followed the same protocol in all years. Of all samples we measured the pH (CaCl_2_) and determined the C:N ratio. A detailed description of the method can be found in Appendix S4

#### 2.2.6 Climate and deposition data

Air temperature and precipitation were measured at the plateau of the study site in a clearing area at 900 m a.s.l. with a standard meteorological station since 1993. Climate data of the nearby meteorological station Reichraming (approximately 5 km distance) and linear regression models (*R* ^2^ = 0.97, *p* < .001 for temperature; and *R* ^2^ = 0.7, *p* < .001 for precipitation) were used to reconstruct the monthly mean temperature and precipitation sums values for the decade preceding the direct on‐site measurements (1983–1993). This earlier period was deemed important because of potential response lags in vegetation changes. N and S deposition data were collected at two intensively measured plots in a spruce‐dominated forest on the plateau (IP I) and in a mixed deciduous forest on the slope (IP II; see Appendix S5 for more details).

### 2.3 Data analysis

All analyses were carried out in the R version 3.1.2 (R Core Team 2014).

#### 2.3.1 Environmental variables

We used two sample *t* tests for detection of changes in all normally distributed environmental variables and Wilcoxon signed‐rank test for nonnormal distributions. Mean annual temperatures (MATs) and mean annual precipitation sums (MAP) of the decades 1983–1992 and 2003–2012 were taken for comparison. Accumulated annual deposition rates were taken for the decades 1994–2003 and 2004–2013. Paired t tests were used to compare the soil C:N ratios and total tree cover of 1993 and 2014, and for comparison of the percentage of tree individuals damaged by ungulate browsing in 2005 and 2014. For total tree cover, we further made separate paired *t* tests for plots with and without disturbance. As soil pH values were not normally distributed, we used Wilcoxon signed‐rank tests to compare the data of the two survey years.

#### 2.3.2 Herb layer

We used univariate and multivariate statistics to compare species and community changes between the survey years 1993, 2005, 2010, and 2014. First, changes in individual species abundances between the first survey and subsequent surveys were tested. Second, plot mean Ellenberg indicator values for light (L), temperature (T), soil moisture (F), soil pH (R), and nutrient availability (N) of the resurveys were compared to the first survey. Third, ordination was applied and dissimilarities in the three‐dimensional community space between the first and the last survey were analyzed with indicator values and path analysis in order to detect the relative influence of environmental variables on changes in herb layer composition.

#### 2.3.3 Species abundance changes

We used paired t tests to detect significant temporal changes of cover values of frequent (occurring in at least 20 plots) herb layer species. For comparison, only plots were taken where the species occurred at least in one of the surveys. If the species was absent in one of the surveys, null was taken as cover value.

#### 2.3.4 Community analyses

Following the recommendations of Diekmann (2003), mean Ellenberg indicator values of light (L), temperature (T), soil moisture (F), soil pH (R), and nutrient availability (N) were calculated for each plot and each survey based on the Ellenberg indicator values of each present vascular plant species. To identify changes between the surveys, we used paired *t* tests.

For the Ellenberg R and N values for which related, directly measured soil data were available, we conducted correlation tests with Pearson's moment correlation coefficient (Ellenberg N ~ C:N ratio) and Spearman's rank correlation (Ellenberg R ~ pH) to test for representativeness. Similarly, we used hemispherical measurements of forest floor radiation from the year 1993 (Mayer et al., 2013) to conduct Spearman's rank correlation with Ellenberg L values. As only a limited number of measured soil temperature together with Ellenberg T values (four plots) and measured soil moisture together with Ellenberg F values (six plots) existed, we only compared them qualitatively (see Appendix S1 for details of measurement methods).

We calculated the Sørensen index, its fraction of nestedness, and the Simpson dissimilarity using betapart 1.3 (Baselga, 2010). We used a *t* test after 100 times of resampling 50% of the vegetation data points to compare the indices of the resamples from 2014 and 1993.

We used nonmetric multidimensional scaling (nMDS) of the R package *vegan 2.2‐1* with a dissimilarity matrix of Bray–Curtis distances to evaluate alterations in community composition over time (Oksanen et al., 2015). As dissimilarity matrix we took the herb layer data of the 143 plots of all surveys (1993, 2005, 2010, 2014) that resulted in a total of 572 plots. nMDS is an ordination method which iteratively searches the best representation of a multidimensional dissimilarity matrix in a predefined low‐dimensional space. The less dissimilar the plots are in their species composition, the closer they are positioned in the nMDS space. The stress value indicates the quality of this representation: The lower the value, the better the data are represented in the nMDS space. We chose a three‐dimensional nMDS space and repeated the calculations 200 times with random starting arrangements of plots to find the model with the least stress. We extracted the axis scores and conducted correlation tests of each of the axis scores and the mean Ellenberg indicator values using Pearson's moment correlation coefficient to find the environmental variables prescribed by the nMDS axis scores. We further subtracted the axis scores of the plots sampled in 1993 from the paired plots sampled in 2014 to calculate the difference vectors (∆ axis scores). These vectors represent the magnitude and direction of change in species composition over time along prescribed environmental variables. We used Ellenberg indicator values to describe these variables. We applied MANOVA (Pillai–Bartlett test statistic) with the three ∆ axis scores as response: first without a grouping variable to test whether there was a significant change in species composition in the three‐dimensional nMDS space and subsequently with disturbance as grouping variable to test whether there was an impact of disturbance on this change. We used permutational multivariate analysis of variance (PERMANOVA, Anderson 2001) of the R package *vegan 2.2‐1* (Oksanen et al., 2015) with Euclidean distances and 10,000 permutations to detect correlations between the three ∆ axis scores and the ∆ of the mean Ellenberg values and disturbance variable (*n* = 143). We further used the ∆ axis scores to calculate Euclidean distance vectors for each point, representing the movement of each plot in the three‐dimensional space between the survey years 1993 and 2014. The length of these vectors depicts the magnitude of species composition change. We implemented these vectors as variable for the herb vegetation in a path model to test the influence of local environmental variables (Ellenberg indicator values R, F, and T, disturbance, browsing, tree and shrub cover) on these alterations. Following Grace, Anderson, Olff, and Scheiner (2010), we conceived an initial model combining the variables with pathways of influence based on our theoretical knowledge (Figure 1). For all environmental variables (including Ellenberg indicator values), except disturbance, we calculated ∆ values by subtracting the data of 1993 from those of 2014. As our variable for the herb layer consists only of positive values that represent the magnitude of change and not its direction, we transformed the negative ∆ values of the environmental variables to positive ones to obtain undirected variables. Disturbance was used as binary variable, with 1 = “disturbance occurred” and 0 = “no disturbance occurred” on the plot. We standardized all continuous variables to make them comparable for the analysis (Schielzeth, 2010). Considering the nonnormal distribution of the data, we conducted correlation tests using Spearman's rank correlation. Environmental variables that were significantly correlated with the herb layer variable were selected and implemented in the path model (Table 1). Pathways were added or removed if they improved the model and were also biologically meaningful. For the path analysis we used the *sem* function of the *lavaan* package (version 0.5–20) with maximum‐likelihood estimation, robust standard errors, and Satorra–Bentler scaled test statistic (Rosseel, 2012).

**Table 1:** Correlations of environmental variables and changes in herb layer composition. Only variables with significant (*p* < .05, bold) correlation and variables significantly correlated with them were implemented in the path model

∆Herb layer composition (Spearman's rank correlation coefficient)	*r* ^2^	*p*
|∆ Browsing|	.03	.768
|∆ Conifers|	.16	.056
|∆ shrub|	.10	.247
|∆ Tree layer cover|	.39	**<.001**
Disturbance	.11	.203
|∆N|	.06	.473
|∆F|	.27	**.001**
|∆R|	.26	**.002**
|∆T|	.21	**.012**

## 3 Results

### 3.1 Changes in climate, deposition, and soil chemistry

Macroclimate, as measured in a clearing area in the study site, airborne S deposition, and soil chemistry were subject to significant shifts between 1983–1992 and 2004–2013 (MAT), 1994–2003 and 2004–2013 (S deposition), and 1993 and 2014 (pH, Table 2): MAT increased, while S deposition decreased significantly, whereas MAP and N deposition did not exhibit any significant trends. Results of the soil analysis reveal a significant increase in soil pH, whereas the decrease in the soil C:N ratio was not significant.

**Table 2:** Temporal trends of climate, deposition, and soil parameters at the study site Zöbelboden. Significant *p* values (*p* < .05) are given in bold. MAPs and MATs of each year of the decade before the first and last vegetation survey were taken for comparison (*n* = 10). For the deposition analyses the rates of the first 10 years of deposition measurements were compared with the last 10 years (*n* = 10) as no older records were available

Climate	*n*	1983–1992	*SE*	2004–2013	*SE*	∆	*p*
MAP [mm]	10	1547.86	162.17	1606.51	260.80	58.65	.555
MAT [°C]	10	6.91	0.51	7.83	0.50	0.92	**.001**

Deposition	*n*	1994–2003	*SE*	2004–2013	*SE*	∆	*p*
N deposition IP1 [kg ha^−1^ year^−1^]	10	18.79	3.81	17.00	2.89	−1.79	.218
N deposition IP2 [kg ha^−1^ year^−1^]	10	12.51	1.97	11.41	1.81	−1.10	.315
S deposition IP1 [kg ha^−1^ year^−1^]	10	8.27	3.12	4.49	1.09	−3.78	**<.001**
S deposition IP2 [kg ha^−1^ year^−1^]	10	5.28	1.03	3.54	0.64	−1.75	**<.001**

Soil parameters	*n*	1993	*SE*	2014	*SE*	∆	*p*
pH (CaCl_2_)	49	6.17	0.79	6.25	1.00	0.08	**.042**
C:N ratio	49	14.89	3.14	13.92	3.37	−0.97	.104

### 3.2 Changes in tree layer cover

We did not detect a significant change in total tree cover across the study area (*p* = .40) and on undisturbed plots (*p* = .12) but a significant decrease (*p* < .01) from 77 ± 33% in 1993 to 57 ± 32% in 2014 on all disturbed plots (*n* = 49).

### 3.3 Changes in ungulate browsing

Percentage of juvenile tree species that were damaged by browsing increased significantly (*p *<* *.001) from 35 ± 25% in 2005 to 55 ± 26% in 2014.

### 3.4 Cover changes of individual species

**Figure 2:**
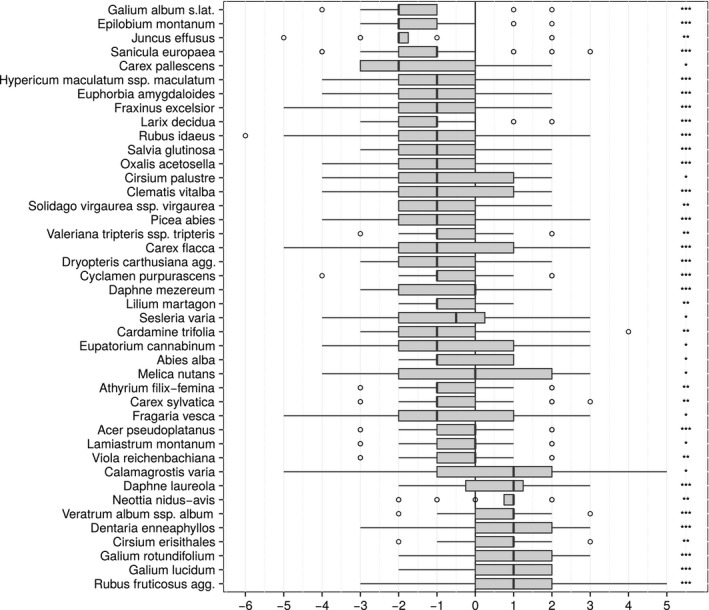
Significant abundance changes of vascular plant species with at least 20 occurrences in the plots (*n* = 143). Asterisks symbolize the level of significance (*p < .05, **p < .01, ***p < .001). Nine species increased significantly, while 33 species decreased in cover

Of the total of 275 species that were found in the surveys, 82 occurred in at least 20 plots. Thereof, nine species increased significantly in cover, whereas 33 decreased (*p* < .05) (Figure 2).

### 3.5 Overall changes in the herb layer

**Figure 3:**
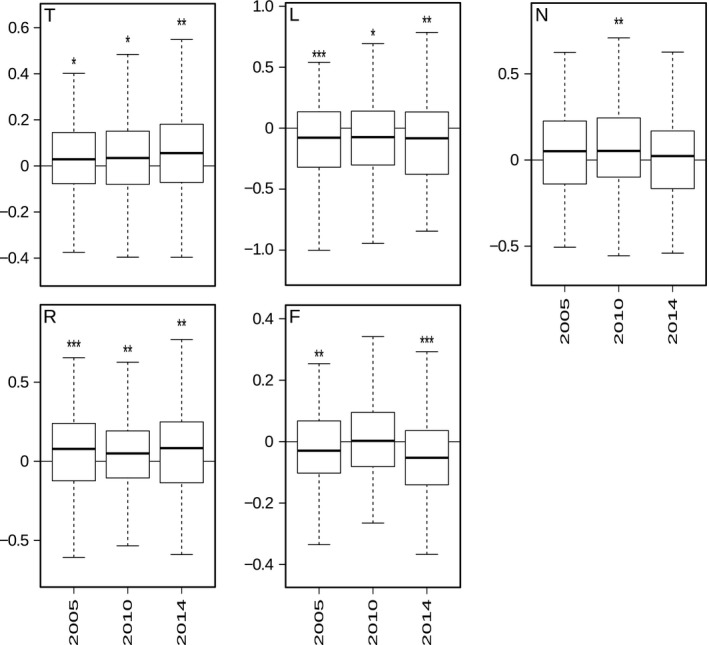
Boxplots of the deviation of unweighted mean Ellenberg community indicator values of 2005, 2010, and 2014 from the first survey in 1993. The community indicator values represent means of the individual Ellenberg indicator values of each vascular plant species occurring per plot (*n* = 143). Asterisks symbolize the level of significance (*p ****<** **.05, **p ****<** **.01, ***p ****<** **.001)

Mean herb layer cover decreased from 63.3 ± 30.9% to 52.1 ± 28.4% (*p* < .001). The total number of vascular plant species occurring in the herb layer of the 143 plots dropped from 235 in 1993 to 207 in 2014. The Sørensen index increased significantly from 0.0023 to 0.0028 (*p* < .001). The Simpson dissimilarity of the all plots increased significantly from 0.9357 to 0.9373 (*p* < .001), whereas nestedness decreased from 0.021 to 0.017 (*p* < .001). All Ellenberg indicator values except for the N value exhibited significant changes between the first and the last survey (Figure 3). Temperature (T) and soil pH (R) values increased, whereas light (L) and soil moisture (F) values decreased.

The correlation test between Ellenberg N value and C:N ratio showed no significant correlation (*n* = 49, ρ = −0.01, *p *=* *.935), whereas Ellenberg R was significantly related to pH (*n* = 49, ρ = 0.68, *p *<* *.001; see Appendix S1, Fig. S1). The relationship between measured forest understory radiation and Ellenberg L value was significant (*n* = 51, ρ = 0.32, *p *=* *.018; see Appendix S1, Fig. S2). Measured annual maximum and mean soil temperature at four plots located in climatically divergent sites in the study area (different altitudes and slope aspects) increased with the Ellenberg T value, yet not significantly due to the low power of the test (*n* = 4, ρ ≥ 0.6, *p *≤* *.417), while the minimum and mean temperature did not (see Appendix S1, Fig. S3). Measured soil moisture (mean, maximum, and minimum) at these and two additional plots of the year 2014 did not relate with Ellenberg F value (*n* = 6, ρ < −0.46, *p* > .321; see Appendix S1, Fig. S4).

### 3.6 Changes in plant species composition between plots over time

**Figure 4:**
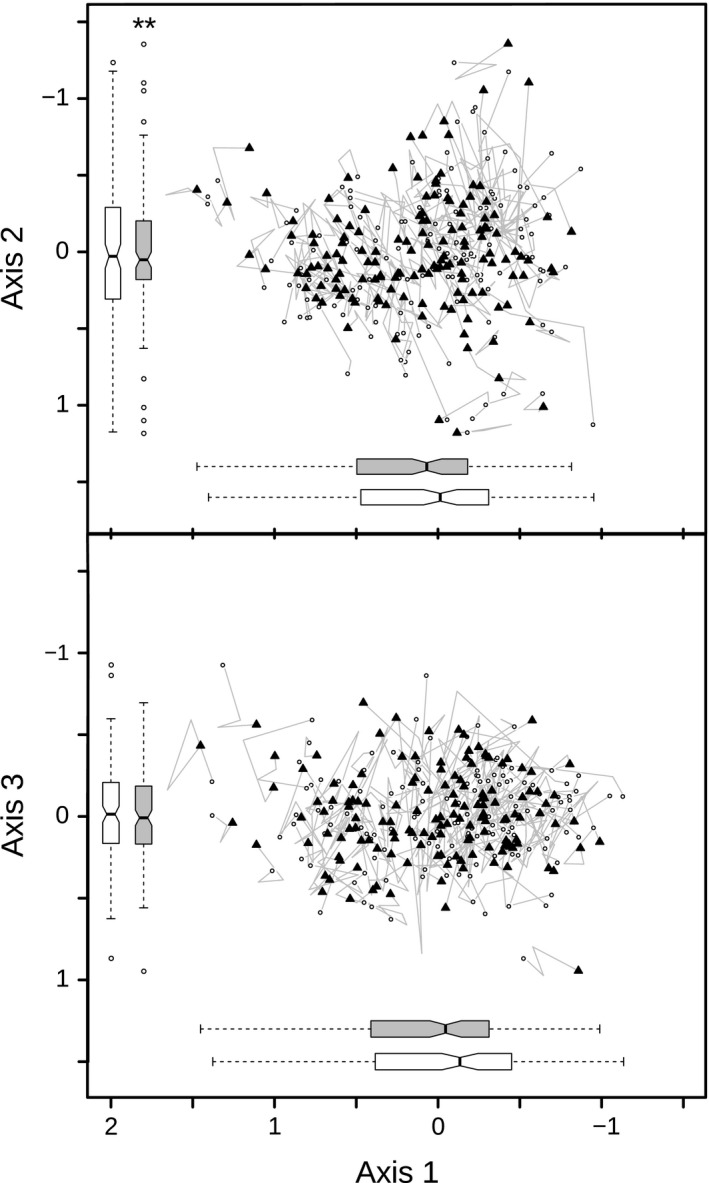
Results of the nonmetric multidimensional scaling ordination plot (stress = 15.8) based on cover data of vascular plant species in plots (*n* = 143) of the herb layer: White dots represent the plot data from 1993, black filled triangles those from 2014. The gray paths indicate the movement of plots between the surveys. The 2005 and 2010 plot data were taken as vertices for the paths. The boxplots depict the variance of the survey years 1993 (white) and 2014 (gray). Asterisks symbolize whether there was a significant change in homogeneity of variance (*p < .05, **p < .01, ***p < .001)

The three‐dimensional nMDS ordination explains 79% of the variation of species composition between plots and has a stress value of 0.158 (Figure 4). According to the widely accepted classification of Clarke (1993), nMDS ordinations with stress values <0.2 are considered to deliver an acceptable representation of the original distance matrix. nMDS axis 1 is significantly correlated (*p* < .05) with all Ellenberg indicator values, nMDS axis 2 with all but N, and nMDS axis 3 with R and L (Table 3). The variance in axis 2 scores decreased significantly (*p* < .01) from 1993 to 2014. Results of the MANOVA analysis reveal a substantial overall change in species composition (MANOVA without grouping variable: Pillai = 0.123, *F*_3,140_ = 6.55, *p* < .001) and a significant impact of disturbance on the magnitude of this change (MANOVA with disturbance as grouping variable: Pillai = 0.136, *F*_3,139_ = 7.28, *p* < .001).

**Table 3:** Correlations of the axes of the nonmetric multidimensional scaling ordination and the calculated mean Ellenberg indicator values. Significant correlations (*p* < .05) are given in bold

Mean Ellenberg indicator value	Axis 1	Axis 2	Axis 3
Soil nitrogen (N)	−**0.80**	0.02	−0.04
Soil moisture (F)	−**0.74**	−**0.39**	−0.06
Light (L)	**0.73**	−**0.45**	−**0.18**
Soil pH (R)	**0.65**	**0.43**	−**0.26**
Temperature (T)	−**0.26**	**0.11**	−0.08

### 3.7 The factors that shape changes in species composition

As revealed by the PERMANOVA analysis (Table 4; for the full table see Appendix S2), changes in tree cover explained most of the shifts of plots in the nMDS ordination (*F *=* *116.886, *p* < .001). Further, changes in mean N (*F *=* *20.314, *p* < .001) and R (*F *=* *12.902, *p* < .001) values showed highly significant correlations with the ∆ axis scores. Ellenberg F (*F *=* *6.910, *p *=* *.001) and T (*F *=* *5.251, *p* = .008) had lower, but also significant influence, whereas disturbance (*p* = .156) was not significantly correlated.

**Table 4:** Results of the PERMANOVA analysis of the ∆ axis values of the nMDS in relation to changes in tree layer cover, nutrient availability (N), soil pH (R), soil moisture (F), temperature (T), and disturbance. *df* = degree of freedom, SS = sum of squares, *F *= *F* value per permutation. Bold *p* values indicate significant values (*p* < .05). *p* values are based on 10,000 permutations

	*df*	SS	*F*	R^2^	*p*
∆ tree layer cover (∆TLC)	1	11.513	116.886	0.338	**<.001**
∆ nutrient availability (∆N)	1	2.001	20.314	0.059	**<.001**
∆ soil pH (∆R)	1	1.271	12.902	0.037	**<.001**
∆ soil moisture (∆F)	1	0.681	6.910	0.020	**.001**
∆ temperature (∆T)	1	0.517	5.251	0.015	**.008**
Disturbance	1	0.177	1.793	0.005	.158

**Figure 5:**
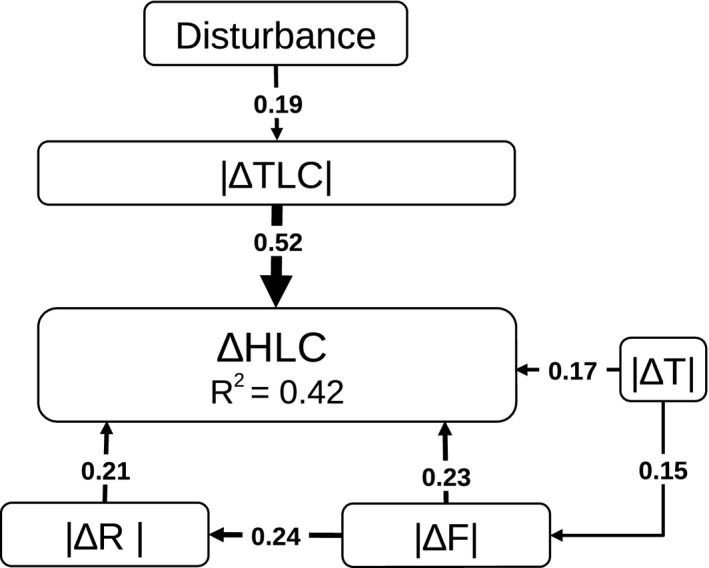
The final model of the path analysis (χ^2^ = 5.2, *df* = 7, *p* = .64) depicts the correlations between the different environmental variables and herb layer changes over time. Arrows represent relationships between variables and are labeled with standardized path coefficients. Thicker arrows symbolize stronger correlations. *R* ^2^ gives the proportion of variance explained by the correlated variables. Full parameters of the pathway analysis are given in Table 3

The temporal trends of the environmental variables implemented in the path model are represented in Table 5. Whereas average tree layer cover remained stable at the study site, the Ellenberg indicator values T, F, and R changed significantly: Temperature increased from 4.67 ± 0.26 to 4.72 ± 0.24 (*p* < .01) and soil reactivity from 6.71 ± 0.52 to 6.78 ± 0.49 (*p* < .01), whereas soil moisture decreased from 5.25 ± 0.26 to 5.20 ± 0.25 (*p* < .001). The final path model (Figure 5) fitted the data well with χ^2^ = 5.2, *df* = 7, *p* = .64. Note that *p* values >.05 indicate that there is no significant deviation between data and the model. All pathways given in the model are significant (*p* < .05). Changes in tree layer cover (|∆TLC|) had a major impact on the herb layer, whereas changes in Ellenberg indicator values for soil pH (|∆R|), soil moisture (|∆F|), and temperature (|∆T|) were also significantly correlated with the herb layer, explaining 43% of the total variation of the herb layer composition changes (∆HLC) (Table 6).

**Table 5:** Changes of the environmental variables that were implemented in the path model (*n* = 143) between 1993 and 2014. Note that for the path analysis, only absolute ∆ values were used. Significant *p* values are given in bold. For disturbance, only the number of plots that were affected by disturbance is given

Environmental variables	1993	*SE*	2014	*SE*	∆	*p*
∆ tree layer cover (∆TLC)	71.62	34.33	68.66	30.80	−2.96	.402
∆ temperature (∆T)	4.67	0.26	4.72	0.24	0.04	**.005**
∆ soil moisture (∆F)	5.25	0.26	5.20	0.25	−0.06	**<.001**
∆ soil pH (∆R)	6.71	0.52	6.78	0.49	0.07	**.008**
Disturbance			49	*Plots affected*		

**Table 6:** Parameters of the path analysis describing the relationships between the implemented variables (∆HLC, |∆TLC|, |∆R|, |∆F|, |∆T|, disturbance): Estimate represents unstandardized path coefficients, *z* is the test statistic (estimate divided by standard deviation), and *p* (>|*z*|) is the probability under the null hypothesis of no relationship between the variables. Std. Coeff. are coefficients standardized by standard deviation

Pathway	Estimate	*z*	*p* (>|*z*|)	Std. Coeff.
∆ herb layer composition (∆HLC)				
← |∆ tree layer cover| (|∆TLC|)	0.51	6.04	<.001	0.52
← |∆ soil pH| (|∆R|)	0.21	2.71	.007	0.21
← |∆ soil moisture| (|∆F|)	0.22	3.09	.002	0.23
← |∆ temperature| (|∆T|)	0.16	2.31	.021	0.17
|∆ tree layer cover| (|∆TLC|)				
← disturbance	0.19	2.18	.029	0.19
|∆ soil moisture| (|∆F|)				
← |∆ temperature| (|∆T|)	0.15	2.11	.035	0.15
|∆ soil pH| (|∆R|)				
← |∆ soil moisture| (|∆F|)	0.24	2.57	.010	0.24

## 4 Discussion

### 4.1 Changes of the herb layer and of environmental parameters

Our analysis revealed a directional change over time in forest floor species composition (which was more pronounced in disturbed than in undisturbed plots), a decrease in species richness and an increase in the dissimilarities between plots. These shifts can be explained by the interaction of large‐scale environmental changes (climate change, air pollution) with local pressures that affect plots differently (primarily tree canopy changes). These results are robust insofar as observer errors have been kept low (Hülber et al., 2008), major environmental changes have been measured in situ, and because changes detected by plant indicator analysis were supported by their relations with measured data.

Among the directional changes, a recovery from soil acidification seemed to be most apparent. We did not only observe an increase in the Ellenberg R value but also directly measured an increase in soil pH. In addition, we showed that Ellenberg R values correspond with soil pH measurements. Acid‐tolerant species (e.g. *Juncus effusus* L., *Hypericum maculatum* Crantz) are found among the species that declined in cover, whereas neutral or basiphilous species increased (e.g. *Galium lucidum* All., *Cirsium erisithales* Scop., *Calamagrostis varia* Host). This finding corroborates results from an earlier analysis in the study site (Hülber et al., 2008) and also reflects the results of more recent studies in temperate forest ecosystems, which had been exposed to acid deposition (Reinecke et al., 2014; Vanhellemont, 2014). Although the soils of the study area, having carbonate bedrock, are well buffered to acid deposition, topsoils might have been affected by acidification and currently may be in recovery after sulfate deposition decreased considerably. As part of the area was used as a pasture until around 1900, increasing base saturation during forest succession may play a role together with the fact that most forest soils in Austria were acidified because of overuse and related base cation depletion over centuries (Jandl et al., 2012).

Further, we found signs that forest vegetation responded to an increase in temperatures with a relative increase in more thermophilic plants. Moreover, we observed a decrease in the Ellenberg F value, suggesting that soils within the study area became drier. Species adapted to wet soils declined (*Cirsium palustre* L., *Juncus effusus*), whereas species restricted to drier and warmer conditions increased (e.g. *Galium lucidum*,* Daphne laureola* L.). Although mean annual precipitation sums did not change significantly during the last three decades, increased temperatures, particularly in the form of more frequent and extended heat waves (APCC 2014), may have caused higher evapotranspiration rates and hence drier soils. The Ellenberg indicator values for temperature, which indicate thermophilization, were positively related to annual soil temperature maxima, which were measured at four plots in the study area representing the main climatic conditions as to elevation and slope aspects. Although we could not show a relationship between Ellenberg F values and measured soil moisture, which stem from only six plots and covered a relatively humid year, we hypothesize that the particularly dry springs and summers in the years 2003, 2007, 2011, and 2013 have caused the observed decrease in mean Ellenberg F values. First, Ellenberg F values seem to be mostly correlated with summer drought (Schaffers & Sýkora, 2000), and second, large parts of the study area are characterized by shallow soils above dolomite bedrock, with low water‐holding capacity. Hartl‐Meier et al. (2015) by using tree‐ring isotopes observed a significant reduction in tree growth during several drought events in the last decades. Their study underpins that moisture limitation may play a role for changes of the forest understory even in this relatively humid mountain climate.

The results of the PERMANOVA reveal that changes in tree cover were strongly related to changes in the composition of the herb layer. Further, they show that nutrient availability (N), soil pH (R), soil moisture (F), and temperature (T) are independent factors that were influencing the vegetation of the herb layer. Similarly, the path analysis indicates that thermophilization and drying are not mere artifacts from an increase in disturbance gaps, rendering sites more open to radiation and soil warming, but independent drivers of compositional changes in the herb layer. This confirms the results of previous works (Hedwall & Brunet, 2016; Küchler et al., 2014; Lenoir, Gégout, Dupouey, Bert, & Svenning, 2010; Savage & Vellend, 2015) which found indications of a climate driven floristic change in the understory of European forests. Canopy closure due to decreasing tree harvest and tree aging, which has the potential to buffer thermophilization of forest floor vegetation (De Frenne et al., 2013), is less significant in the study area because mature forest stands, where canopy closure is near its maximum level, predominate. We also think that the shift from cooler and more humid to warmer and drier conditions triggered the decline in species richness in the study area. Mostly species that could be found on the cool and moist end of the gradient have disappeared (e.g. *Carex demissa* Hornem., *Dryopteris expansa* (C. Presl) Fraser‐Jenk. & Jermy, *Cardamine flexuosa* With.). This species loss could probably not be compensated by new species adapted to warmer and drier conditions due to dispersal limitations or a lack of drought‐adapted species in the regional species pool. Yet overall, disturbances might have prevented even stronger species loss via the observed increase in the variation in site conditions among plots. In plots affected by disturbance, tree layer cover decreased, its cooling (sensu De Frenne et al., 2013) and shading effects weakened, enabling new species which are better adapted to warmer temperatures to colonize the forest floor (Overpeck, Rind, & Goldberg, 1990). However, the strength of the influence of disturbance on the warming and drying signal of the forest floor vegetation is not fully clear. An improved assessment of this relationship would need precise measurements of disturbance intensities. In a relatively small disturbance gap (<200 m²) in the study area, no change in the average daily soil temperature has been observed when compared to closed forests (Kobler, Jandl, Dirnböck, Mirtl, & Schindlbacher, 2015). These authors also observed that soils in cleared gaps are wetter due to less evapotranspiration from trees. For European beech forests, Leuschner and Lendzion (2009) showed that air humidity and soil moisture are the most important factors in limiting certain forest plant species to occur. Considering future climate predictions (APCC 2014), drought events will probably extend its range and become more frequent. As gap size and structure strongly control microclimatic changes (Ritter, Dalsgaard, & Einhorn, 2005) and frequency and strength of disturbances are predicted to increase in European forests (Seidl et al., 2014), these factors may become even more important for future compositional changes of forest ecosystems.

Annual wet N deposition rates in canopy throughfall were within the range of empirical critical loads of 10–20 kg N ha^−1^ year^−1^ (Bobbink et al., 2010), but N deposition exceeds this threshold when dry and occult deposition is taken into account (Mayer et al., 2013). In a prior analysis of forest floor vegetation changes in the study area, Hülber et al. (2008) have reported a homogenization in the species composition from acid, wet sites and base‐rich, dry sites toward more intermediate conditions. They related this trend to eutrophication in response to N deposition. However, it appears that more frequent disturbance events disrupted this relationship since 2005 and that the influence of climate change became more important as described in the next sections of the discussion. The change in the soil C:N ratio did not provide an indication of increased N availability for plant growth, nor did the results based on Ellenberg N values. High rates of N mineralization and shallow soils with high preferential flow rates causing major loss of N via leaching (Jost, Dirnböck, Grabner, & Mirtl, 2011) render the study site relatively insensitive to N deposition. At least in the short to medium term, N deposition effects on forest plants seem to be weak in sites without major N limitation, even when N loads exceed the critical load (Dirnböck et al., 2014).

**Figure 6:**
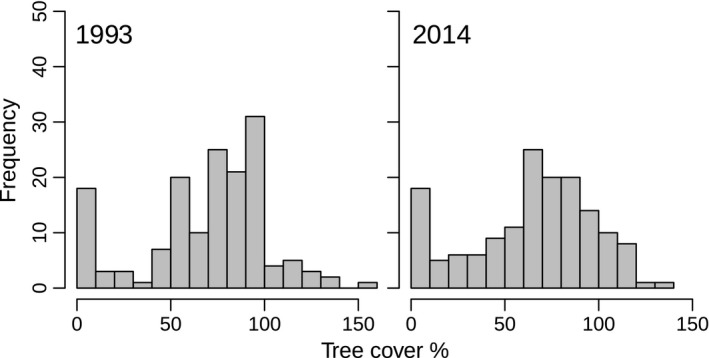
Frequency of estimated tree covers in 1993 and 2014, categorized to 10 percent classes. The tree cover was calculated by summarizing the estimated cover values of each tree layer

The decrease in the Ellenberg L value, which was significantly related to forest understory radiation measured at a subset of the plots, suggests that light availability on the forest floor decreased. This might also have caused the observed reduction in the herb layer cover. However, we did not detect an increase in overall canopy cover which might be due to the methodology (see discussion below). Nevertheless, Diekmann (2003) recommends the application of unweighted Ellenberg indicator values in species‐rich communities. Yet in forest ecosystems a weighted Ellenberg L value might better represent the light conditions of the forest floor (Diekmann, 2003). In an additional analysis we compared weighted mean Ellenberg L values of 1993 and 2014 and could not find a significant change between the two surveys. However, the herb layer cover decreased significantly in contrast to the stable tree cover. We argue that disturbances are responsible for these contrary trends: One‐third of the plots were affected by disturbances between 1993 and 2014, mainly by windthrow and the removal of bark beetle‐infested trees. As mentioned above, tree layer cover on the affected plots decreased and light availability for the herb layer increased, likely shaped by disturbance gap size (Canham et al., 1990). On the other hand, canopies likely became denser on the majority of plots not affected by disturbances and this may have led to a decrease in the herb layer cover. The latter is a general trend in many Central European forests because timber extraction has declined during the last decades (Verheyen et al., 2012). Accordingly, we found a shift in the frequency of tree layer cover classes from dominance of plots with medium canopy closure to more evenly distributed frequencies of different tree cover classes (Figure 6). We consider changes in irradiance the main control for herb layer cover at our site. Apart from irradiance, N deposition can also increase forest floor biomass (Gilliam et al., 2016). In fact, it has been shown that soil C:N ratios, usually lowered by N deposition, showed a negative relationship with forest floor herb and grass cover in the study site (Diwold, Dullinger, & Dirnböck, 2010). Nevertheless, this effect might have been too weak because C:N ratios decreased only slightly. Drier soils, as indicated by the Ellenberg F value, may have additionally contributed to the decrease in the herb layer cover. One additional factor controlling herb layer cover is the variability in the shrub cover. More open tree canopies benefit juvenile tree growth and therefore increase shading of understory plants. This is apparent in parts of the study area, but in others ungulate browsing is strongly limiting tree regeneration.

### 4.2 Relative contributions of drivers to herb layer changes

We conducted a path analysis to identify the relative contributions of environmental drivers to changes in the herb layer composition. Our hypothesis was that disturbance is the most important factor as it controls canopy closure and thus has severe indirect impact on the light regime and microclimate on the forest floor. We measured the intensity of compositional change by calculating the three‐dimensional distance in the nMDS ordination for each plot between the surveys of 1993 and 2014. Hence, we implemented absolute values of change of our variables that caused a reduction in correlation coefficients. However, the results of the path analysis give a good representation of the relative contribution of the most important drivers of compositional changes in the herb layer.

Our results support changes in tree cover as the major driver of forest floor vegetation changes and the regulative effect of disturbances. The influence of disturbance was lower than expected, yet its relative importance likely is underrepresented as we did not account for intensity (e.g. relative reduction in trees) and gap size of disturbance. The impact of disturbance on changes in forest floor plant communities may also differ between sites within the study area because contrasting plant functional types occur on the relatively moist plateau soils and at dry slopes, that is plants investing in resource acquisition versus resource conservation which thus are differently adapted to cope with forest disturbance (Seebacher, Dirnböck, Dullinger, & Karrer, 2012). However, disturbance has a key role as it can rapidly provoke fundamental changes in the environmental conditions of the herb layer. As the intensity and gap size of disturbances are spatially highly variable, disturbances enhance site variability by generating patches with different light and nutrient availability and may thus have promoted the increase in dissimilarity and the decrease in nestedness between plots. Soil pH, soil moisture, and temperature were significantly related to compositional changes in the herb layer and likely reflect local variation in site conditions, but also the influence of regional environmental shifts (like decreased S deposition, drought events and increased temperature). Disturbances pave the way for immigration of new species that are better adapted to modified environmental conditions as they temporarily remove the inhibiting effect of light limitation of the canopy on forest floor communities (Overpeck et al., 1990). The relative influence of soil moisture, soil pH, and temperature in the path model is difficult to disentangle as the implemented mean Ellenberg indicator values considerably correlate. The PERMANOVA results suggest a stronger influence of changes in the nutrient availability (N value). Yet, this has to be interpreted carefully as the calculated mean N values were not correlated with the measured soil C:N ratio, which did not change significantly. It is very likely that the mean N value is a better indicator for total productivity and may integrate other factors such as moisture availability or disturbance (Schaffers & Sýkora, 2000). However, our results show that soil pH has a significant influence on the shifts in the herb layer. Given the high correlation between mean Ellenberg R value and measured soil pH, both in our study and in work from others (Diekmann, 2003; Schaffers & Sýkora, 2000), changes in soil pH did at least moderately drive species shifts in the herb layer. In addition, our results support that floristic changes in the herb layer were independently influenced by temperature and soil moisture changes. We did not detect an influence of ungulate browsing on changes in herb layer composition, although browsing damage increased substantially over time. Yet, the direction and intensity of browsing influence on plant communities are very variable and may further vary between distinct understory plant communities (Hobbs, 1996). Hence, the signal might have been too weak for detection. Moreover, the path analysis revealed no significant correlation between tree cover changes and changes of soil parameters and temperature, although other studies already highlighted influences of tree layer cover on soil conditions and microclimate (De Frenne et al., 2013; Leuschner & Rode, 1999). Furthermore, the relative contribution of individual drivers may be moderated by differences in site characteristics between plots (Bernhardt‐Römermann et al., 2015). Besides, site history (e.g. former land use) and initial site conditions like soil nutrient status may also play an important role in shaping responses of temperate forest communities to environmental changes (Burton, Mladenoff, Clayton, & Forrester, 2011; Müllerová et al., 2015; Naaf & Kolk, 2016).

## 5 Conclusions

We identified direct and indirect effects of multiple environmental drivers on forest floor vegetation changes in a forest that has recently been affected by strong disturbances (windthrow, bark beetle outbreaks). Such disturbances are predicted to increase in future with intensifying climate change. Our study may therefore serve as a showcase of the likely trajectories of forest vegetation changes in future. We found that the relative importance of environmental factors affecting changes in forest understory composition has shifted over time from N deposition to climate‐related processes. Yet, further observation and modeling are necessary to better understand forest floor vegetation changes in a disturbance‐prone future.

## Conflict of Interest

None declared.
