# Supplementary material for: Multiple environmental changes drive forest floor vegetation in a temperate mountain forest
Source: Ecol Evol. 2017 Mar 1;7(7):2155–68. doi: 10.1002/ece3.2801 (PMC5383490; doi:10.1002/ece3.2801)
Supplement: Supplementary file 1 [file ECE3-7-2155-s001.docx]

**Appendix S1** *Relationship between Ellenberg values and measured data*

**Methods**

Plot mean Ellenberg values were compared with measured data. For soil chemistry and light conditions the corner point plots of the 100x100 m grid (excluding rock outcrops and forests younger than 20 years) were used, totalling to 49 for soil chemistry sampling (paired samples in 1993 and 2014) and 51 for light measurements (measurement year 2005). The methods for soil pH and soil C:N ratio calculation are described in detail in Appendix S4. We used radiation estimates from hemispherical photographs taken at 1 m height in the year 2005 (see Mayer et al. 2013) in order to establish the relationship of measured radiation at the forest understory and Ellenberg L values. Since climate station data only captures the macroclimatic conditions we also analysed an existing soil temperature and moisture data set for microclimatic conditions under the tree canopy. The locations of these plots were chosen to represent the main climatic conditions within the study area, i.e. along altitudinal gradients and different slope aspects. For soil temperature we used continuous three hourly measurements in 5 cm depth (iButton, Thermochron, n=5) between 2008 and 2014 and calculated the annual mean, maximum and minimum values. These measurements were available for 4 plots where Ellenberg T values could be calculated for the same period and were located at the plateau and at slopes with different aspects in the study area. Soil moisture data was measured monthly between May and November during the year 2015 at 6 plots where Ellenberg F values could be calculated. Four of these plots were the same as for the soil temperature measurements.

rho = -0.01

*P* = 0.935

rho = 0.68

*P* < 0.001

Figure S1. Scatterplot of measured soil pH and Ellenberg R value (left) and of soil C:N ratio and mean Ellenberg N value (right). n=49 (mean values of the years 1993, 2005, 2014). Spearman correlation coefficient is shown.

rho = 0.32

*P* = 0.018

Figure S2. Scatterplot of measured radiation at the forest understorey (July and August 2005) and mean Ellenberg L values from 51 plots. The significant (p < 0.05) Spearman correlation coefficient is shown in this plot.

rho = 0.6

*P* = 0.417

rho = -0.2

*P* = 0.917

rho = 0.8

*P* = 0.333

Figure S3. Scatterplot of measured minimum, mean and maximum annual soil temperature (5 cm depth, continues measurements during the years 2008 to 2014) and mean Ellenberg T values from 4 plots.

rho = -0.44

*P* = 0.381

rho = -0.49

*P* = 0.321

rho = -0.49

*P* = 0.321

Figure S4. Scatterplot of measured minimum, mean and maximum soil moisture (5-10 cm depth, measured during the snow-free season in the year 2015) and mean Ellenberg F values at 6 plots.
