# Supplementary material for: Multiple environmental changes drive forest floor vegetation in a temperate mountain forest
Source: Ecol Evol. 2017 Mar 1;7(7):2155–68. doi: 10.1002/ece3.2801 (PMC5383490; doi:10.1002/ece3.2801)
Supplement: Supplementary file 2 [file ECE3-7-2155-s002.docx]

**Appendix S2** *PERMANOVA results*

Results of the PERMANOVA analysis of the ∆ axis values of the NMDS in relation to changes in tree layer cover (∆ TLC), nutrient availability (∆ N), soil pH (∆ R), soil moisture (∆ F), temperature (∆ T) and disturbance and their interactions. Df = degree of freedom, SS = sum of squares, *F* = F value per permutation. Bold P-values indicate significant values (*P* < 0.05). P-values are based on 10000 permutations.

|  | Df | SS | *F* | *R^2^* | *P* |
| --- | --- | --- | --- | --- | --- |
| ∆ TLC | 1 | 11.513 | 116.886 | 0.338 | **< 0.001** |
| ∆ N | 1 | 2.001 | 20.314 | 0.059 | **< 0.001** |
| ∆ R | 1 | 1.271 | 12.902 | 0.037 | **< 0.001** |
| ∆ F | 1 | 0.681 | 6.910 | 0.020 | **0.001** |
| ∆ T | 1 | 0.517 | 5.251 | 0.015 | **0.008** |
| disturbance | 1 | 0.177 | 1.793 | 0.005 | 0.158 |
| ∆ TLC x ∆ N | 1 | 0.095 | 0.960 | 0.003 | 0.373 |
| ∆ TLC x ∆ R | 1 | 0.129 | 1.309 | 0.004 | 0.262 |
| ∆ N x ∆ R | 1 | 0.139 | 1.409 | 0.004 | 0.233 |
| ∆ TLC x ∆ F | 1 | 0.125 | 1.268 | 0.004 | 0.269 |
| ∆ N x ∆ F | 1 | 0.176 | 1.785 | 0.005 | 0.152 |
| ∆ R x ∆ F | 1 | 0.210 | 2.131 | 0.006 | 0.113 |
| ∆ TLC x ∆ T | 1 | 0.118 | 1.201 | 0.003 | 0.295 |
| ∆ N x ∆ T | 1 | 0.344 | 3.497 | 0.010 | **0.036** |
| ∆ R x ∆ T | 1 | 0.017 | 0.172 | 0.000 | 0.887 |
| ∆ F x ∆ T | 1 | 0.499 | 5.066 | 0.015 | **0.008** |
| ∆ TLC x disturbance | 1 | 0.057 | 0.576 | 0.002 | 0.574 |
| ∆ N x disturbance | 1 | 0.155 | 1.572 | 0.005 | 0.196 |
| ∆ R x disturbance | 1 | 0.390 | 3.960 | 0.011 | **0.024** |
| ∆ F x disturbance | 1 | 0.114 | 1.158 | 0.003 | 0.307 |
| ∆ T x disturbance | 1 | 0.247 | 2.508 | 0.007 | 0.079 |
| ∆ TLC x ∆ N x ∆ R | 1 | 0.094 | 0.957 | 0.003 | 0.364 |
| ∆ TLC x ∆ N x ∆ F | 1 | 0.708 | 7.190 | 0.021 | **0.002** |
| ∆ TLC x ∆ R x ∆ F | 1 | 0.074 | 0.750 | 0.002 | 0.468 |
| ∆ N x ∆ R x ∆ F | 1 | 0.039 | 0.401 | 0.001 | 0.703 |
| ∆ TLC x ∆ N x ∆ T | 1 | 0.067 | 0.682 | 0.002 | 0.502 |
| ∆ TLC x ∆ R x ∆ T | 1 | 0.067 | 0.678 | 0.002 | 0.505 |
| ∆ N x ∆ R x ∆ T | 1 | 0.064 | 0.650 | 0.002 | 0.523 |
| ∆ TLC x ∆ F x ∆ T | 1 | 0.048 | 0.485 | 0.001 | 0.637 |
| ∆ N x ∆ F x ∆ T | 1 | 0.225 | 2.287 | 0.007 | 0.102 |
| ∆ R x ∆ F x ∆ T | 1 | 0.141 | 1.435 | 0.004 | 0.227 |
| ∆ TLC x ∆ N x disturbance | 1 | 0.165 | 1.679 | 0.005 | 0.174 |
| ∆ TLC x ∆ R x disturbance | 1 | 0.008 | 0.080 | 0.000 | 0.961 |
| ∆ N x ∆ R x disturbance | 1 | 0.130 | 1.323 | 0.004 | 0.256 |
| ∆ TLC x ∆ F x disturbance | 1 | 0.060 | 0.606 | 0.002 | 0.559 |
| ∆ N x ∆ F x disturbance | 1 | 0.243 | 2.468 | 0.007 | 0.083 |
| ∆ R x ∆ F x disturbance | 1 | 0.365 | 3.701 | 0.011 | **0.030** |
| ∆ TLC x ∆ T x disturbance | 1 | 0.123 | 1.246 | 0.004 | 0.277 |
| ∆ N x ∆ T x disturbance | 1 | 0.445 | 4.521 | 0.013 | **0.013** |
| ∆ R x ∆ T x disturbance | 1 | 0.063 | 0.636 | 0.002 | 0.538 |
| ∆ F x ∆ T x disturbance | 1 | 0.074 | 0.750 | 0.002 | 0.470 |
| ∆ TLC x ∆ N x ∆ R x ∆ F | 1 | 0.134 | 1.358 | 0.004 | 0.253 |
| ∆ TLC x ∆ N x ∆ R x ∆ T | 1 | 0.171 | 1.737 | 0.005 | 0.167 |
| ∆ TLC x ∆ N x ∆ F x ∆ T | 1 | 0.052 | 0.525 | 0.002 | 0.614 |
| ∆ TLC x ∆ R x ∆ F x ∆ T | 1 | 0.117 | 1.186 | 0.003 | 0.292 |
| ∆ N x ∆ R x ∆ F x ∆ T | 1 | 0.136 | 1.376 | 0.004 | 0.235 |
| ∆ TLC x ∆ N x ∆ R x disturbance | 1 | 0.011 | 0.114 | 0.000 | 0.935 |
| ∆ TLC x ∆ N x ∆ F x disturbance | 1 | 0.161 | 1.638 | 0.005 | 0.172 |
| ∆ TLC x ∆ R x ∆ F x disturbance | 1 | 0.110 | 1.115 | 0.003 | 0.321 |
| ∆ N x ∆ R x ∆ F x disturbance | 1 | 0.212 | 2.153 | 0.006 | 0.115 |
| ∆ TLC x ∆ N x ∆ T x disturbance | 1 | 1.092 | 11.082 | 0.032 | **< 0.001** |
| ∆ TLC x ∆ R x ∆ T x disturbance | 1 | 0.058 | 0.587 | 0.002 | 0.577 |
| ∆ N x ∆ R x ∆ T x disturbance | 1 | 0.801 | 8.137 | 0.024 | **0.002** |
| ∆ TLC x ∆ F x ∆ T x disturbance | 1 | 0.084 | 0.855 | 0.002 | 0.419 |
| ∆ N x ∆ F x ∆ T x disturbance | 1 | 0.137 | 1.392 | 0.004 | 0.239 |
| ∆ R x ∆ F x ∆ T x disturbance | 1 | 0.099 | 1.009 | 0.003 | 0.354 |
| ∆ TLC x ∆ N x ∆ R x ∆ F x ∆ T | 1 | 0.123 | 1.254 | 0.004 | 0.280 |
| ∆ TLC x ∆ N x ∆ R x ∆ F x disturbance | 1 | 0.017 | 0.169 | 0.000 | 0.890 |
| ∆ TLC x ∆ N x ∆ R x ∆ T x disturbance | 1 | 0.038 | 0.384 | 0.001 | 0.714 |
| ∆ TLC x ∆ N x ∆ F x ∆ T x disturbance | 1 | 0.077 | 0.785 | 0.002 | 0.453 |
| ∆ TLC x ∆ R x ∆ F x ∆ T x disturbance | 1 | 0.059 | 0.596 | 0.002 | 0.555 |
| ∆ N x ∆ R x ∆ F x ∆ T x disturbance | 1 | 0.263 | 2.673 | 0.008 | 0.072 |
| ∆ TLC x ∆ N x ∆ R x F x ∆ T x disturbance | 1 | 0.151 | 1.535 | 0.004 | 0.207 |
| Residuals | 79 | 7.781 |  | 0.228 |  |
| Total | 142 | 34.062 |  | 1 |  |
