# Supplementary material for: Multiple environmental changes drive forest floor vegetation in a temperate mountain forest
Source: Ecol Evol. 2017 Mar 1;7(7):2155–68. doi: 10.1002/ece3.2801 (PMC5383490; doi:10.1002/ece3.2801)

**Appendix S3** *Results of abundance changes of individual plant species*

Figure S5-8. Significant changes of Braun-Blanquet cover abundance classes of vascular plant species with at least 20 occurrences in the plots (n = 143). Asterisks symbolize the level of significance (* p<0.05, ** p<0.01, *** p<0.001). The species are sorted by their change in abundance in 2014 compared to the first survey in 1993, with species that decreased most shown first, and species that increased most shown last. The different colors of the boxplots represent the abundances of the resurveys in 2005, 2010 and 2014 compared to the baseline data (1993).


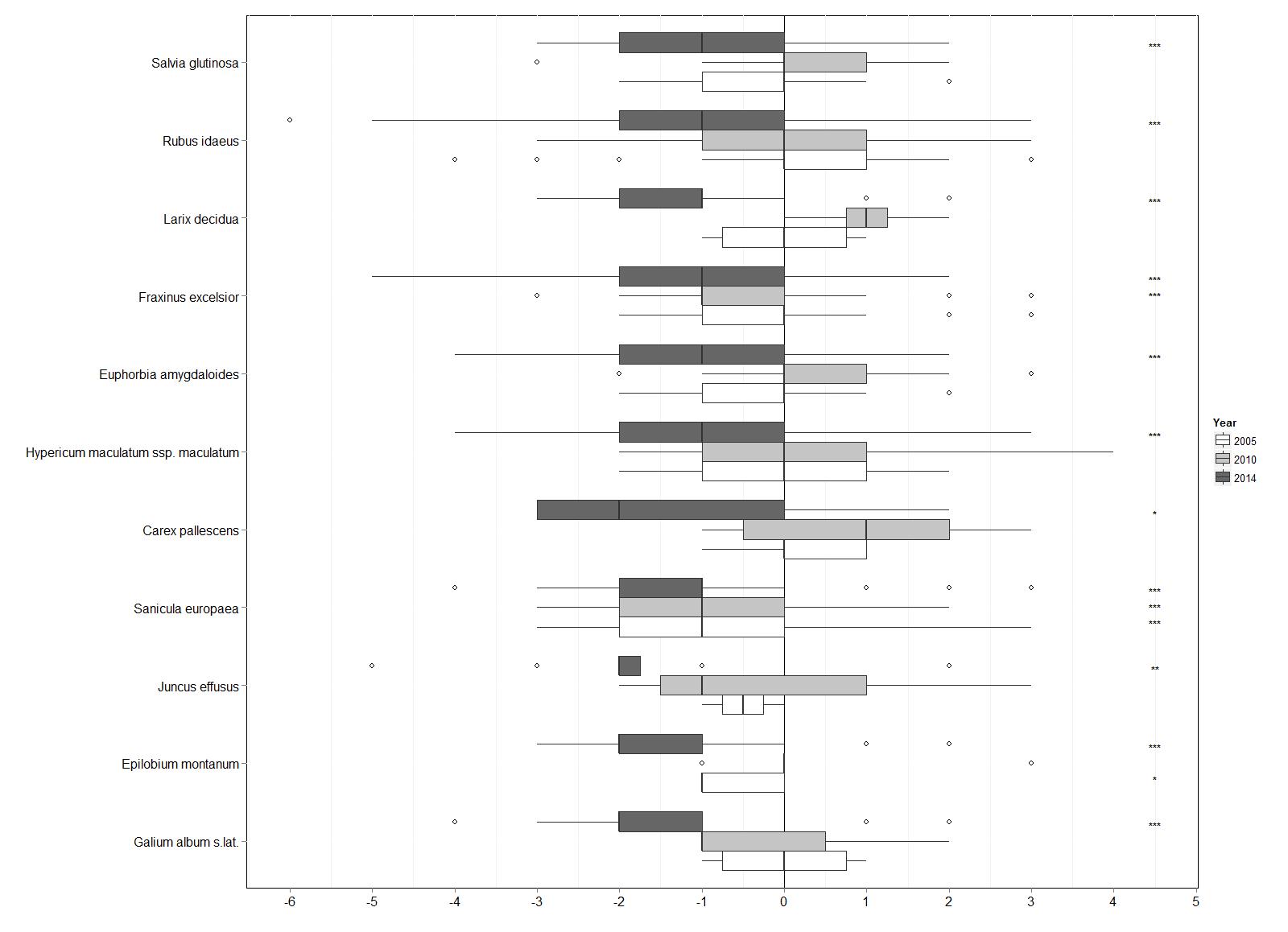


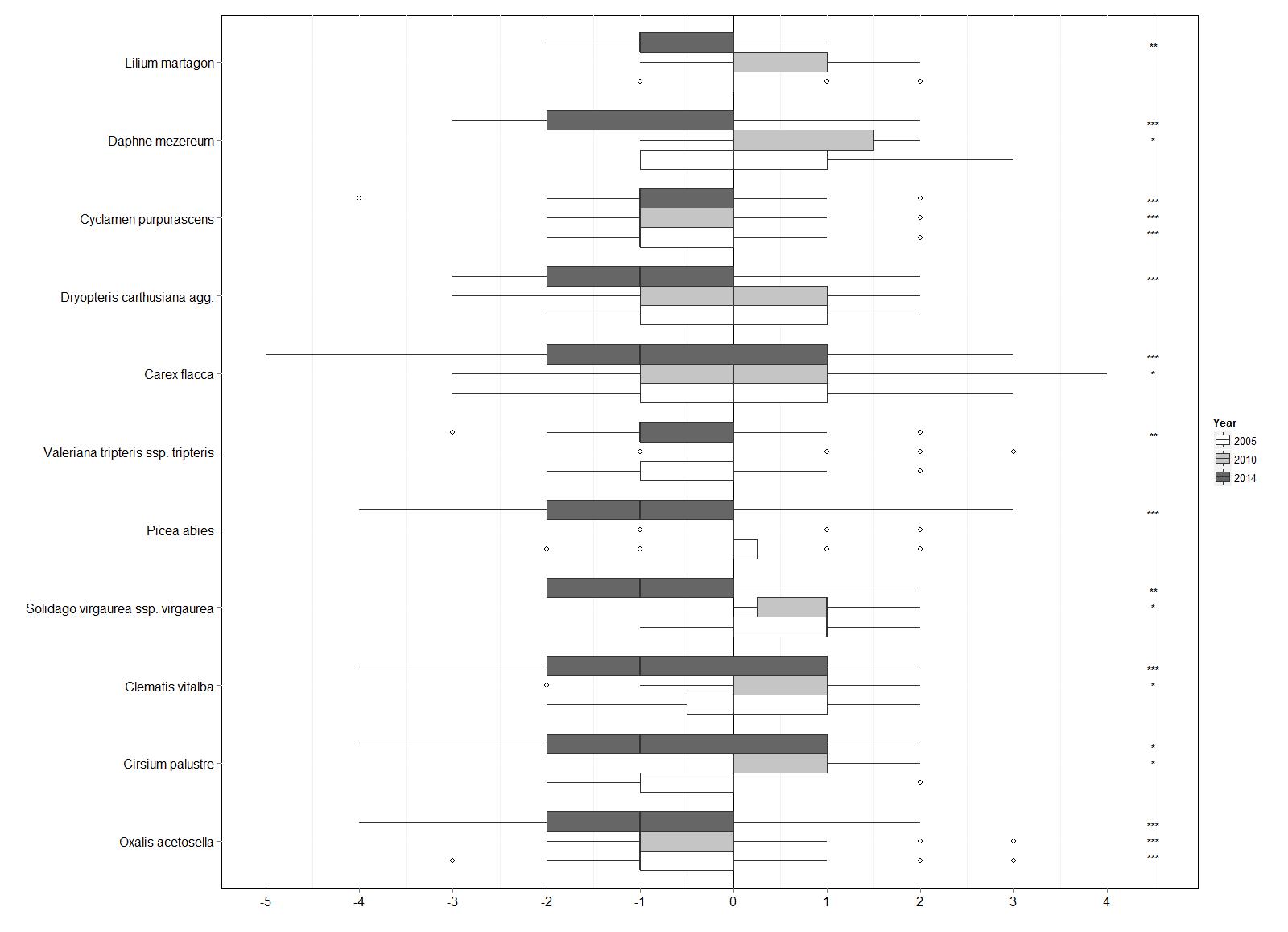

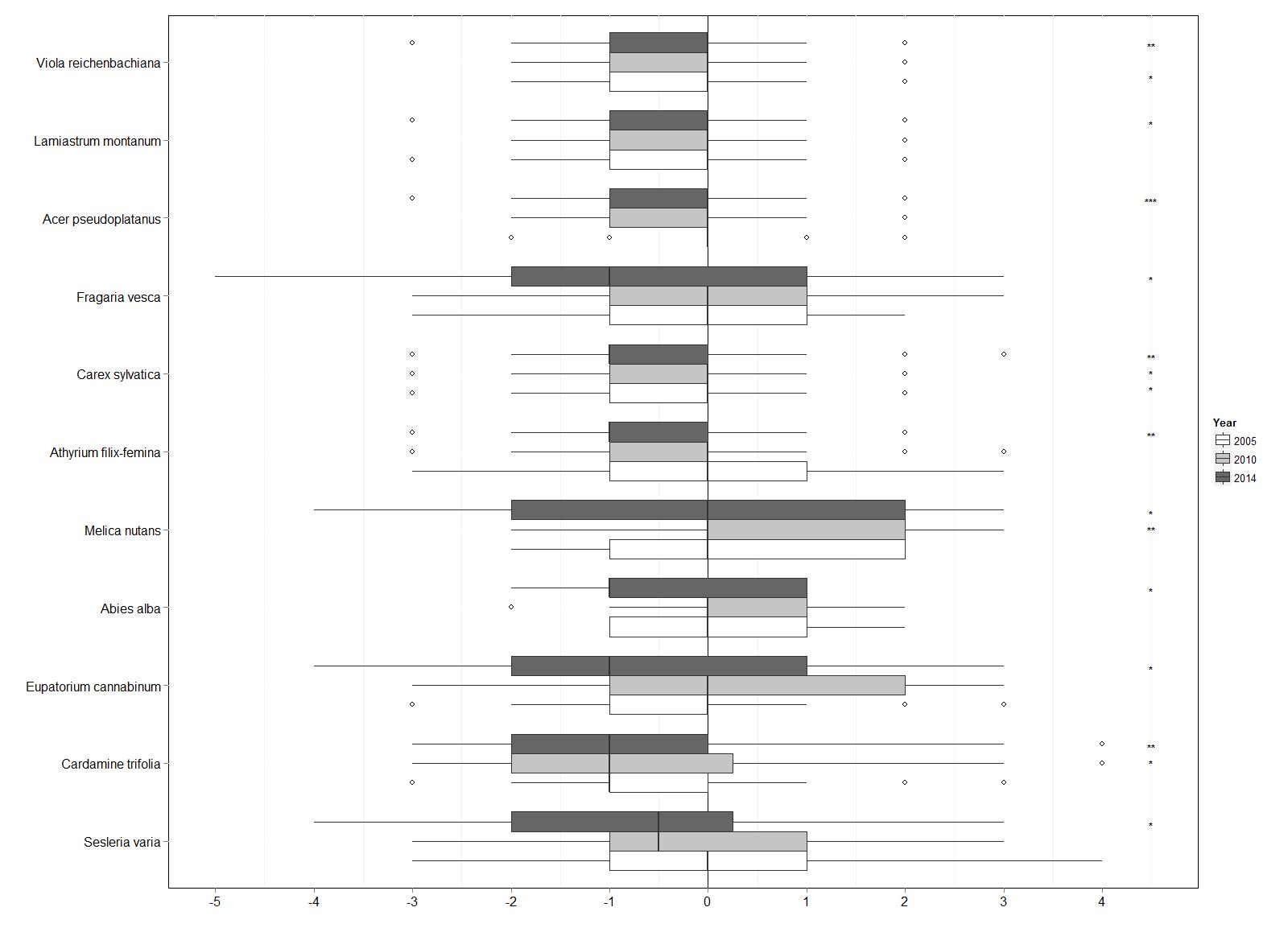


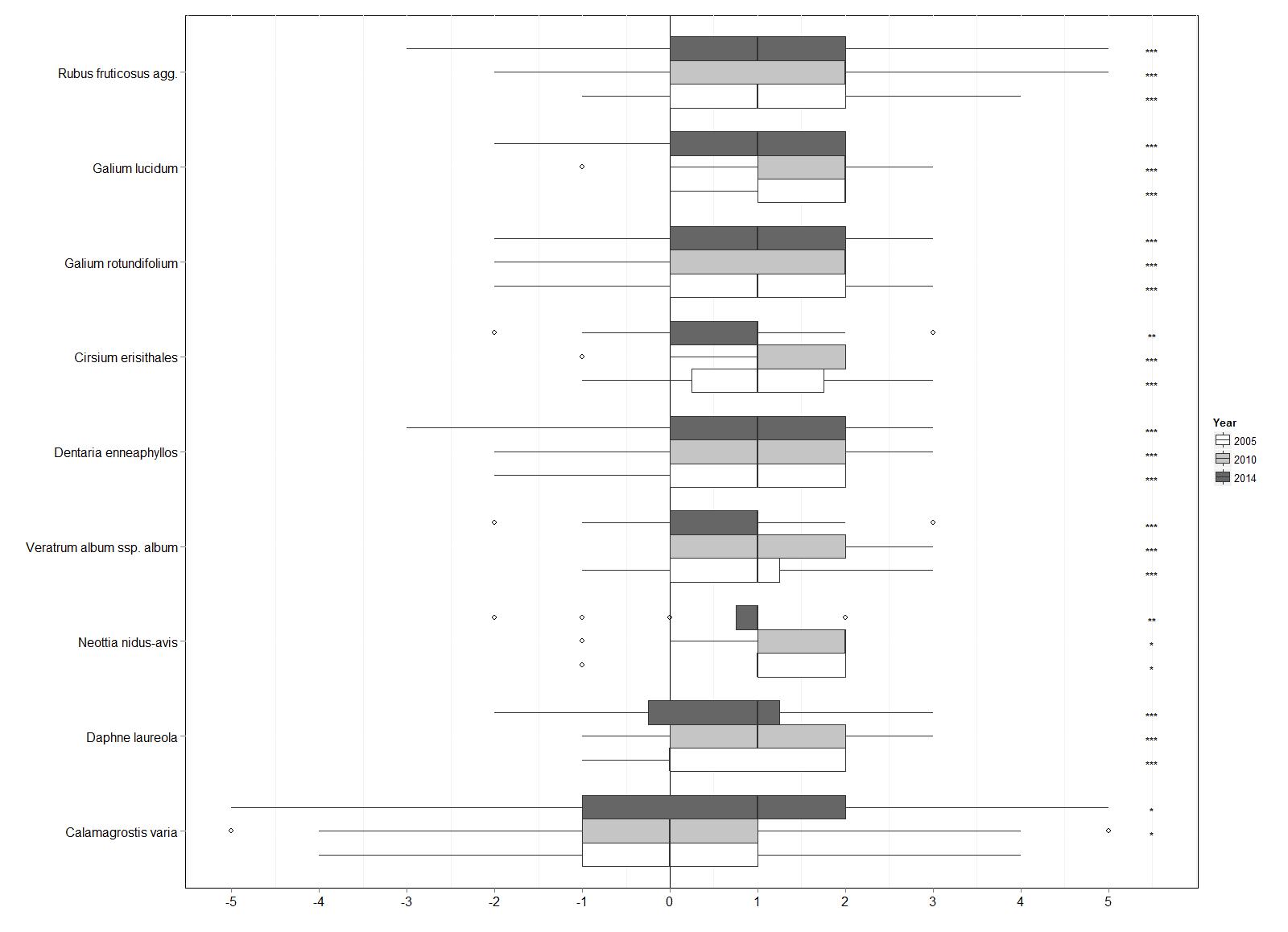

Supplement: Supplementary file 3 [file ECE3-7-2155-s003.docx]
