# Supplementary material for: Multiple environmental changes drive forest floor vegetation in a temperate mountain forest
Source: Ecol Evol. 2017 Mar 1;7(7):2155–68. doi: 10.1002/ece3.2801 (PMC5383490; doi:10.1002/ece3.2801)
Supplement: Supplementary file 4 [file ECE3-7-2155-s004.docx]

**Appendix S4** *Detailed method description for the soil data*

We collected mixed soil samples at 49 plots in July and August 2014 for comparison with soil data from 1992 and 2004. Sampling followed the same protocol in all years. These plots comprise every second permanent vegetation plot (excluding disturbed plots, rock outcrops, clear-cut areas and young plantations of less than 20 years). For each mixed soil sample, three soil cores of 4 cm diameter were taken from the upper mineral soil layer (0-10 cm) after litter removal. Soil cores were taken in 4.5, 5.0 and 5.5 m distance to the permanent plots, with 2.5 m distance to the 1992 and 2004 sampling sites. In all surveys, soil samples were dried at approximately 30°C, coarse aggregates crushed and dried again until weight remained constant. Samples were then sieved through a 2 mm sieve. Soil suspensions were made by dissolving 5 g of each soil sample in 12.5 ml of 0.01 M CaCl_2_ solution and pH was measured electrochemically with a pH electrode (Metrohm 654 pH meter in 1992 and 2004 and Argus X pH meter in 2014). For determining the C:N-ratio, the soil samples were ground and further decarbonized using 3 M HCl solution. Organic carbon content was measured after dry combustion using isotope-ratio mass spectroscopy (IRMS). This method deviates from the analysis in 1992 where total content of organic C (TOC) was calculated by subtracting the total content of CaCO_3_ from the total content of C (TC – TIC). Total content of C (TC) was analyzed by dry combustion (1300°) of the samples in O_2_. Released CO_2_ was measured coulometrically (Ströhlein Coulomat 702 and Si 111/6). Total content of CaCO_3_ (TIC) was measured via addition of HCl and volumetrically determination of the released CO_2_ (Scheibler method). Total N content was determined by a modified Kjeldahl method applied in all survey years. Organic N was converted to NH_4+_ by digestion with H_2_SO_4_ and a catalyst (Kjeldahlterm KT8 Gerhardt). Accumulated NH_4+_ was converted to NH_3_ (distillation) and measured by potentiometric titration. To account for N oxygen compounds, salicylic acid was added prior to digestion.
