# Supplementary material for: Multiple environmental changes drive forest floor vegetation in a temperate mountain forest
Source: Ecol Evol. 2017 Mar 1;7(7):2155–68. doi: 10.1002/ece3.2801 (PMC5383490; doi:10.1002/ece3.2801)
Supplement: Supplementary file 5 [file ECE3-7-2155-s005.docx]

**Appendix S5** *Detailed method description for the deposition data*

Nitrate (NO_3_^-^) and ammonium (NH_4_^+^) as well as sulphate (SO_4_^2-^) in throughfall have been monitored since 1994. No measured data from the study site was available for the preceding decade. Throughfall at each site was measured with 15 regularly distributed bulk deposition samplers (∅=20 cm). From 2006 onwards, 17 samplers were used at the IP I. Water samples were analyzed in weekly intervals up to 1999. From 1999 onwards, samples from two consecutive weeks were mixed (volume weighted) and analyzed biweekly. NO_3_^-^ and SO_4_^2-^ were determined by ion chromatography with conductivity detection (Dionex IC System 4000 I until 2002, thereafter with Dionex IC System Serie DX 500, soil water after 2002 was analyzed with Metrohm IC System 7xx-Serie). Concentration of NH_4_^+^ was analyzed with photometric analysis (Milton-Roy 1201 Spectrophotometer). Total inorganic N was derived from the sum of NO_3_^-^ and NH_4_^+^.
